# Investigation of Using Hyperspectral Vegetation Indices to Assess Brassica Downy Mildew

**DOI:** 10.3390/s24061916

**Published:** 2024-03-16

**Authors:** Bo Liu, Marco Antonio Fernandez, Taryn Michelle Liu, Shunping Ding

**Affiliations:** 1 BioResource and Agricultural Engineering Department, California Polytechnic State University, San Luis Obispo, CA 93407, USA; bliu17@calpoly.edu (B.L.); tliu25@calpoly.edu (T.M.L.); 2 Plant Sciences Department, California Polytechnic State University, San Luis Obispo, CA 93407, USA; mferna36@calpoly.edu; 3 Wine and Viticulture Department, California Polytechnic State University, San Luis Obispo, CA 93407, USA

**Keywords:** hyperspectral, machine learning, downy mildew, *Brassica*, vegetation index

## Abstract

Downy mildew caused by *Hyaloperonospora brassicae* is a severe disease in *Brassica oleracea* that significantly reduces crop yield and marketability. This study aims to evaluate different vegetation indices to assess different downy mildew infection levels in the *Brassica* variety Mildis using hyperspectral data. Artificial inoculation using *H. brassicae* sporangia suspension was conducted to induce different levels of downy mildew disease. Spectral measurements, spanning 350 nm to 1050 nm, were conducted on the leaves using an environmentally controlled setup, and the reflectance data were acquired and processed. The Successive Projections Algorithm (SPA) and signal sensitivity calculation were used to extract the most informative wavelengths that could be used to develop downy mildew indices (DMI). A total of 37 existing vegetation indices and three proposed DMIs were evaluated to indicate downy mildew (DM) infection levels. The results showed that the classification using a support vector machine achieved accuracies of 71.3%, 80.7%, and 85.3% for distinguishing healthy leaves from DM1 (early infection), DM2 (progressed infection), and DM3 (severe infection) leaves using the proposed downy mildew index. The proposed new downy mildew index potentially enables the development of an automated DM monitoring system and resistance profiling in *Brassica* breeding lines.

## 1. Introduction

**Figure 1:**
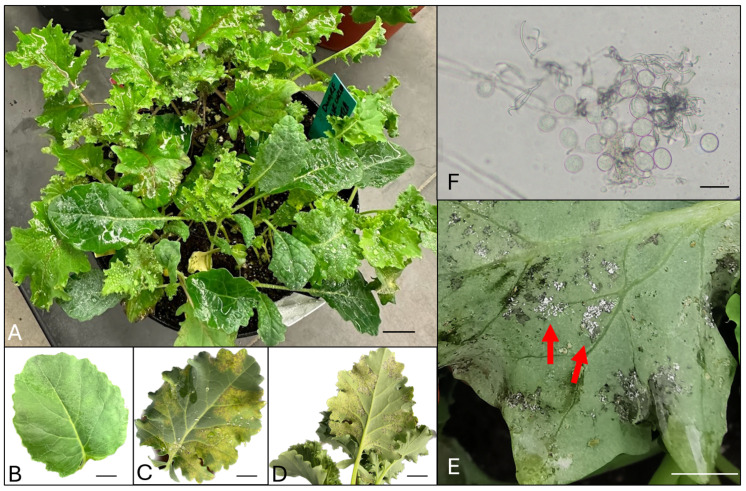
Downy mildew symptoms and signs in *Brassica oleracea*. (**A**). Various varieties of *Brassica oleracea* plants. (**B**). Early infection of downy mildew in a baby kale leaf, note the chlorotic spots and patches. (**C**). Progressed infection of downy mildew in a baby kale leaf, note the chlorotic patches and necrotic webby-like lesions on the upper side of the leaf. (**D**). Progressed infection of downy mildew in a baby kale leaf, note the chlorotic patches and necrotic webby-like lesions on the underside of the leaf. (**E**). A close-up observation of a downy mildew-infected *Brassica* leaf where white sporulation (red arrows) was observed on the underside of the leaf. (**F**). Sporangia and sporangiophore of *H. brassicae* at 100×. (Note: the bars in (**A**–**E**) indicate 1 cm; the bar in (**F**) indicates 100 µm.).

*Brassica oleracea* is a plant species that belongs to the family Brassicaceae, which includes many common vegetable cultivars, such as kale, broccoli, cabbage, cauliflower, and so on. Many vegetables from this species, especially the baby-leafy types, are often used as leafy salads. A foliar disease, downy mildew, caused by *Hyaloperonospora brassicae*, is a major disease in *B. oleracea*. After infection, chlorotic and necrotic spots develop on the leaves (Figure 1), which impact both crop yield and overall plant vitality [1,2], making the leaves unmarketable [3,4]. On the underside of the infected leaves, hyaline sporangiophores are produced, and hyaline sporangia will be produced from the sporangiophores [3]. The sporangia can be disseminated by wind and water splashes and cause epidemics in the field [5,6]. Fungicide application is the major means for downy mildew management, and products have to be applied preventatively. However, early symptoms of downy mildew are hard to identify and can be confused with insect or abiotic damage. 

In the past decade, many studies have been conducted on applying deep learning techniques for the identification and classification of plant foliar diseases using RGB and multispectral datasets [7]. Kanna and Kumar [8] employed deep transfer learning techniques to achieve a 99.90% validation accuracy in automating the detection and classification of three cauliflower diseases (bacterial spot rot, black rot, and downy mildew) using RGB color images. This study only focused on feature extraction from regular RGB color images on adult cauliflower plants, and no other wavelength reflectance attributes were considered. Calderón and Montes-Borrego [9] proposed a multi-spectral and thermal imagery data acquisition system mounted in an unmanned aerial vehicle for downy mildew detection of opium poppy, and the results demonstrated that image-derived canopy temperature and the green/red index were related to physiological stress caused by DM infection. Kundu and Rani [10] presented an automated framework utilizing the Internet of Things and transfer learning for disease prediction in RGB images of pearl millet. The framework attains a 98.78% classification accuracy, offering farmers a cost-effective and efficient tool to improve crop yield and product quality.

Hyperspectral analysis is a non-invasive technique that explores how light interacts with plant tissues across a range of wavelengths. It shows great potential for monitoring the health and physiological status of plants. This technology captures a wide range of spectral bands, from ultraviolet to infrared, enabling precise monitoring of plant responses. It detects subtle variations in spectral signatures, revealing early signs of diseases, nutrient deficiencies, water stress, and other stressors [11], and quantifies crucial parameters like chlorophyll content and photosynthetic efficiency. Therefore, spectral analysis emerges as a potentially valuable tool for evaluating the spectral signatures exhibited by leaves afflicted by DM infection [12]. It also aids in breeding programs and ecological studies, making it a powerful tool for sustainable crop management and ecosystem monitoring, essential for ensuring food security and environmental health.

Numerous studies have demonstrated the effectiveness of hyperspectral data in identifying plant stress factors, including disease, pests, and abiotic stressors such as drought and nutrient deficiencies [13,14]. Hyperspectral remote sensing has been widely employed to assess the nutrient and water status of plants. By analyzing spectral signatures, researchers can detect subtle changes in plant reflectance that occur as a response to stress. For instance, decreased chlorophyll content or altered pigment composition leads to distinct spectral shifts that hyperspectral data can capture [11]. Chlorophyll content, a key indicator of plant health, can be estimated accurately from hyperspectral data [2]. Researchers have developed spectral vegetation indices, such as the Normalized Difference Vegetation Index (NDVI) [15] and the Chlorophyll Index (CI), to quantify chlorophyll levels and photosynthetic efficiency. Similarly, water stress can be detected by analyzing the water absorption features in the near-infrared region. Hyperspectral data acquisition systems mounted on aerial, ground robots, and satellites provide a cost-effective means of large-scale crop monitoring. Beyond agriculture, hyperspectral remote sensing contributes to biodiversity and ecosystem studies, and has been instrumental in understanding the impacts of climate change and land use on ecosystems [16]. With hyperspectral data, researchers can characterize plant species, assess forest health, and monitor changes in vegetation over time [17]. Abdulridha and Ampatzidis [18] applied a multilayer perceptron model to classify hyperspectral vegetation indices for the detection of DM disease in watermelon at several disease severity levels, and the results showed that the proposed method could achieve 86–90% for the laboratory analysis, and 69–91% for the field analysis for severe DM levels. Hernández and Gutiérrez [19] utilized computer vision, hyperspectral imaging, and machine learning to detect early DM disease in grapevine, and an accuracy of 81% was achieved for DM detection. Pithan and Ducati [20] investigated a series of simple ratio vegetation indices using certain selected wavelengths, and the study suggested that wavelengths shorter than 700 nm carry more information than measurements at near-infrared for fungal disease detection on *Vitis vinifera* leaves. Kuswidiyanto and Wang [21] proposed a field-scale system using a hyperspectral camera mounted on an unmanned aerial vehicle and a convolutional neural network to achieve early detection of downy mildew disease in Kimchi cabbage, resulting in an overall accuracy of 0.876 and a 27.07% relative error in estimating disease severity. The literature review shows that hyperspectral data analysis is a very promising method for fast and noninvasive DM detection in plants; however, very few studies have been conducted to evaluate young diseased *Brassica* with vegetation indices, and no study has been done on the exploration of effective wavelengths and DMI development for young *Brassica*.

The objectives of this study are to (1) investigate the influence of DM on the spectral characteristics of these leaves and compare them with the spectral attributes of healthy *Brassica* leaves, (2) evaluate various vegetation indices for assessing different levels of downy mildew (DM) infection in Mildis using hyperspectral data, and (3) develop a downy mildew index by utilizing informative wavelengths extracted from the data to aid in the detection of Brassica downy mildew. Our overarching goal is to contribute to the establishment of a high-throughput and cost-effective phenotyping method. This method has the potential to accelerate the breeding process in *Brassica* production, thereby improving efficiency and productivity in the field. 

## 2. Materials and Methods

### 2.1. Experimental Design

The downy mildew pathogen *Hyaloperonospora brassicae* is an obligate pathogen, and therefore the inoculum needs to be maintained on a living plant. One *H. brassicae* isolate collected from a *Brassica* breeding field in Gilroy, California, was used in this study. The isolate was identified as *H. brassicae* by both the morphology of its sporangia and sporangiophore and the BLASTn of its ITS rDNA and cox2 sequences. A known susceptible *Brassica oleracea* variety Mildis was used to maintain the pathogen and conduct artificial inoculation for hyperspectral data collection. 

**Figure 2:**
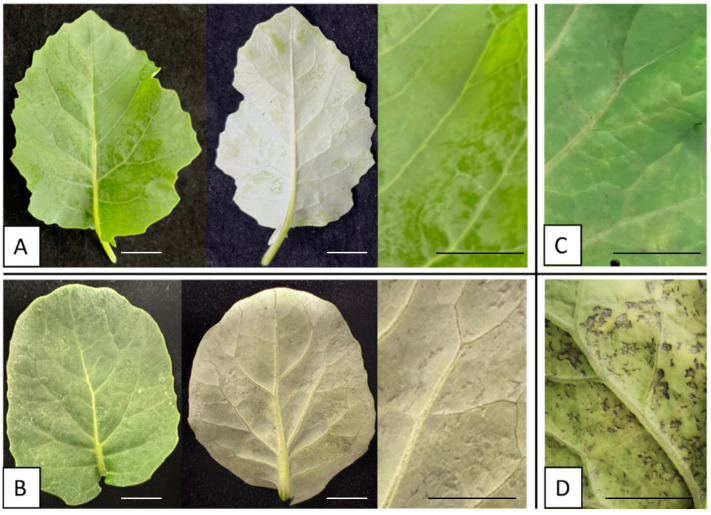
Levels of downy mildew infection on *Brassica oleracea* leaves. (**A**). Healthy leaf with no visual indication of downy mildew infection. (**B**). Early stage of downy mildew infection, where pitted spots can be observed on the lower side of the leaf. (**C**). Progressed downy mildew infection, where pitted spots can be observed from both sides of the leaves and chlorosis developed around the spots. (**D**). Advanced stage of downy mildew infection where lesions can be observed on the leaves. (Note: Bars in (**A**–**D**) indicate 1 cm.).

Artificial inoculation was used to produce downy mildew symptoms. A sporangia suspension was used as the inoculum for the artificial inoculation. To produce the sporangia suspension, sporulating plant leaf tissues were placed in a 50 mL-Falcon tube and submerged in sterile water in the tube. Then, the tube was shaken and vortexed to dislodge sporangia from the plant tissue. Additional sterile water was added to adjust the concentration of sporangia to 10^4^/mL. Then, the sporangia suspension was sprayed on the plants growing in a 72-cell tray using an air brush. A quarter section of the tray was covered during inoculation to keep healthy plants. After inoculation, the inoculated plants were placed in an incubator with 100% RH and 17 °C for 48 h. Then, the plants were incubated on a growth rack (50% RH and 22 °C) for 2 to 14 days to capture different levels of downy mildew disease development. During the 2 weeks of disease development, 3 levels of disease severity are defined and used in the following studies (Table 1, Figure 2). The experiment was repeated three times to capture approximately 100 leaves displaying each level of symptoms.

**Figure 3:**
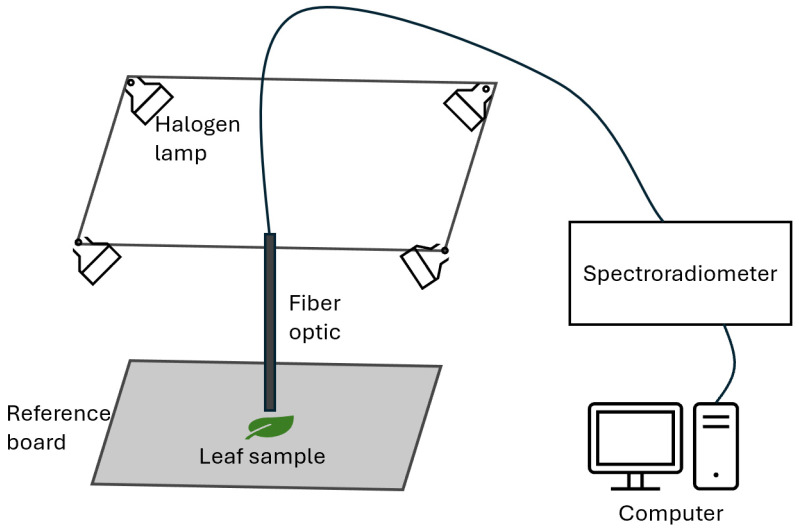
Hyperspectral data acquisition system setup.

An environmentally controlled setup was constructed to ensure uniformity in the spectral measurements. Within this controlled setting, the walls were entirely draped in a material of pure black cloth, serving as an effective barrier to preclude any intrusion of extraneous ambient light, thereby eliminating potential sources of variability. For the attainment of consistent and homogeneous leaf illumination, four Ushio™ Halogen lights (Ushio America, Inc., Cypress, CA) were employed at four corners around the sample, and the light sources were 30 cm from the sample. The instrument employed for measurements was the SVC™ HR-512i spectroradiometer (Poughkeepsie, NY, USA). It was used to take spectral data on targeted small leaf areas for quick and reliable data acquisition. Notably, this spectroradiometer features a 25° field of view (FOV) bare fiber optic cable. The reference board was placed right below the fiber optic cable. One side of the board was a white polytetrafluoroethylene (PTFE) board (Thorlabs Inc., Newton, NY, USA), and it was used for the reference signal capture (without leaf samples), and the other side of the board was a low-reflectance black surface, which was used during the target signal measurement (with leaf samples). The leaf samples were placed 5 mm right beneath the fiber optic sensor, with the downy mildew-infected area directly facing the sensor. The overall hyperspectral data acquisition system is shown in Figure 3. 

### 2.2. Leaf Spectra Acquisition

Spectral radiance measurements spanning the wavelength range of 350 nm to 1050 nm were systematically conducted at the disease development level. Each leaf specimen was meticulously clipped, and measurements were diligently executed up to a duration of 14 days post-inoculation (DPI). The spectral data acquisition process was executed and calibrated using SVC™ HR-1024i Data Acquisition Software version 1.19.3. To guarantee measurement accuracy and precision, the probe underwent calibration procedures before each experimental run. This calibration regimen played a pivotal role in maintaining the exactitude and reliability of the reflectance data acquired during this comprehensive spectral analysis. A total of 100 regions of interest (ROI) representative of healthy leaves and 300 ROI representative of infected leaves, spanning from downy mildew disease level 1 to level 3 (shown in Table 1), were selected for spectral measurements. These measurements were centered within each designated ROI, which was the interveinal area of the leaves where DM infection was observed. Specific considerations were made regarding spectral data under particular circumstances. Namely, spectral data originating from two specific cases were deliberately excluded: (i) Regions of Interest (ROIs) exhibiting scars resulting from external factors other than downy mildew infection, and (ii) leaves displaying chlorosis due to natural senescence. Consequently, a dataset composed of 400 spectra from Mildis leaves with and without DM infections was obtained. 

### 2.3. Spectral Data Processing

**Figure 4:**
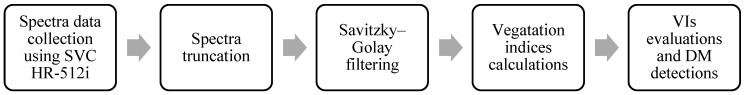
Data analysis flowchart.

To ensure uniformity, comparability, and noise reduction, all spectra were truncated to encompass the wavelength range of 400–950 nm. To enhance data quality, Savitzky–Golay filtering [22] using a second-order polynomial fit with 21 data points was applied to the spectra data. Savitzky–Golay filtering operates by fitting a polynomial function to a small, moving window of data points along the spectrum. It then uses this polynomial to estimate the smoothed values for the data points within the window. This process was repeated for each data point in the spectrum, effectively reducing noise and variability in the data. Once the signals were smoothed, different vegetation indices could be applied, and how well these vegetation indices indicated the DM infection levels could be evaluated. The captured spectra data were executed using MATLAB™ R2023b. The data processing workflow adhered closely to the structured steps outlined in Figure 4.

### 2.4. Vegetation Indices

A Vegetation Index (VI) is a quantifiable metric computed by employing data from two or more designated spectral bands to accentuate the distinctive attributes of vegetation, thereby distinguishing it from other signal sources. The extraction of these spectral attributes is feasible through hyperspectral data analysis, enabling the comparison of indices derived from both healthy and diseased crop specimens, thereby serving as variable inputs [23]. 

#### 2.4.1. VI selection

Some Vegetation Index algorithms have enabled the quantitative assessment of spectral characteristics linked to plant surfaces, aiding in the communication of information related to the physical and chemical attributes of plants. These attributes encompass variables such as water content, compositional makeup, nutrient levels, biomass quantity, and the presence of pathological conditions [24,25]. Following an extensive review of the literature, a set of 37 Vegetation Indices (VIs) has been selected based on their abilities to differentiate plant water content, chlorophyll concentration, and leaf cell structure (Table 2). Subsequently, these selected VIs were employed in the analysis of Mildis spectral data, enabling a comprehensive quantitative assessment of the spectral attributes linked to the physical and chemical characteristics of the plants.

#### 2.4.2. DM Indices Investigation

In addition to the VIs in Table 2, new DM indices were also introduced and analyzed using selected hyperspectral data. The entire hyperspectral data may have a substantial amount of spectral information, but some of the spectral information may not be relevant or redundant for identifying the presence and severity of downy mildew. In this study, the Successive Projections Algorithm (SPA) was used to extract the informative wavelengths to develop DM indices based on these effective wavelengths, and the detailed algorithm information can be found in these references [52,53]. The Successive Projections Algorithm (SPA) is a feature selection technique utilized in hyperspectral data analysis to extract the most informative spectral bands iteratively. Beginning with a dataset containing numerous spectral bands, SPA employs a selection criterion to identify the wavelength that maximally contributes to data separation or variability. The chosen wavelength was added to a growing subset, and the dataset was orthogonalized to ensure subsequent selections were independent. This iterative process continued until a predetermined number of wavelengths, or a stopping criterion, was met. The optimal subset of variables was chosen based on the smallest root mean square error of validation from the multiple linear regression model. In addition, the sensitivity value was employed as an additional measure to explore effective wavelengths that were sensitive to DM infection levels compared to healthy leaves. The sensitivity value was computed by dividing the mean reflectance value of diseased leaves by the mean reflectance value of healthy leaves at each individual wavelength [54,55].

### 2.5. Statistical Analysis and VI Evaluation

To assess the capability of various vegetation indices in distinguishing leaves at distinct levels of infection by downy mildew, Equation (1) was used as the evaluation metric [53]. The M value serves as a quantitative metric for assessing the distinction between the mean values of a VI of two distinct categories, specifically healthy and infected leaves. It normalizes this difference by considering the combined standard deviations of a VI of these categories in Equation (1). A higher M value signifies an improvement in the separation of overlapping data distributions. The M value acts as a discriminant, providing insights into the ability of various vegetation indices to generate data that aids in the effective differentiation between healthy and infected leaves.

(1) $$M=\frac{\left|{Mean}_{Healthy}-{Mean}_{Infected}\right|}{\sigma_{Healthy}-\sigma_{Infected}}$$

To evaluate the best-performing Vegetation Index in discriminating between healthy and infected leaves indicated by M values, three common machine learning classification algorithms, Support Vector Machine (SVM), K-Nearest Neighbor (KNN), and Random Forest, integrated within the MATLAB Statistics and Machine Learning Toolbox™, were employed. 

## 3. Results

**Figure 5:**
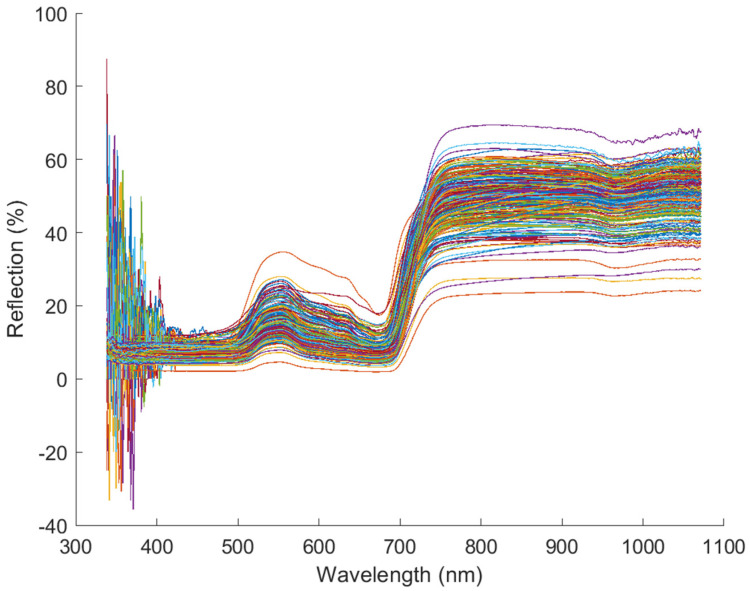
Spectra data at different downy mildew infection development levels. Each waveform represented one sample and was plotted with one color.

Spectra data from 100 typical sample data waveforms with each DM infection level were plotted in Figure 5. Signal data were observed between wavelengths 400 nm and 950 nm, but signals with wavelengths less than 400 nm and higher than 950 nm appeared noisy.

**Figure 6:**
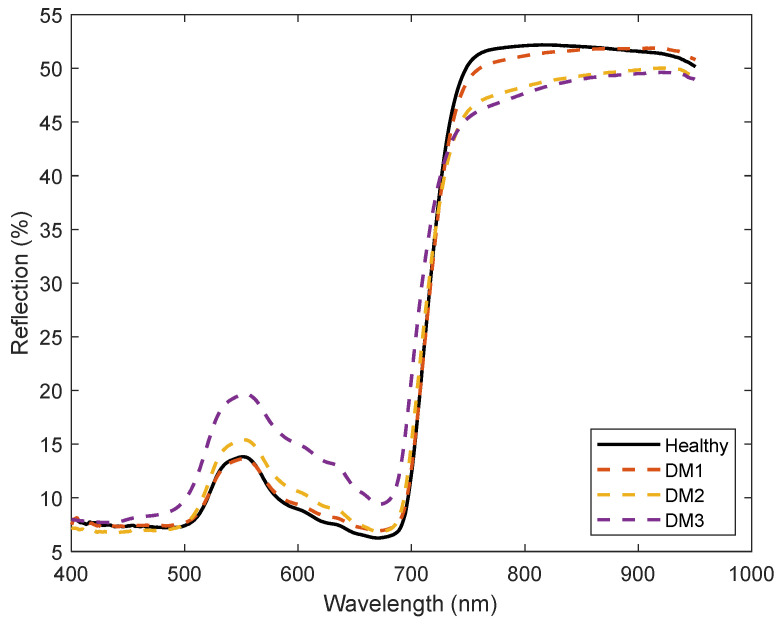
Averaged spectra data for healthy and DM infected leaves.

**Figure 7:**
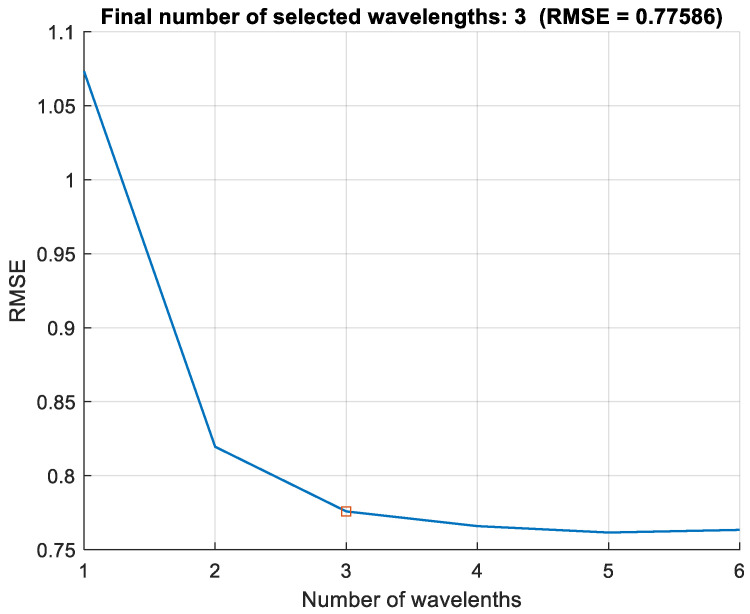
Number of selected wavelengths using SPA.

The averaged spectra curves at different DM infection severity levels are shown in Figure 6. This figure shows light reflections from 400 nm to 950 nm. The reflection percentage of healthy areas consistently remained higher than that of diseased areas across all wavelengths beyond 740 nm. However, intriguingly, this pattern was reversed below the 740 nm threshold. The SPA algorithm was carried out in MATLAB, and the best subset of wavelengths was selected on the basis of the smallest root-mean-square error for the validation after iterative calculations. The plot in Figure 7 ends to level off after three variables are added to the model. 

**Figure 8:**
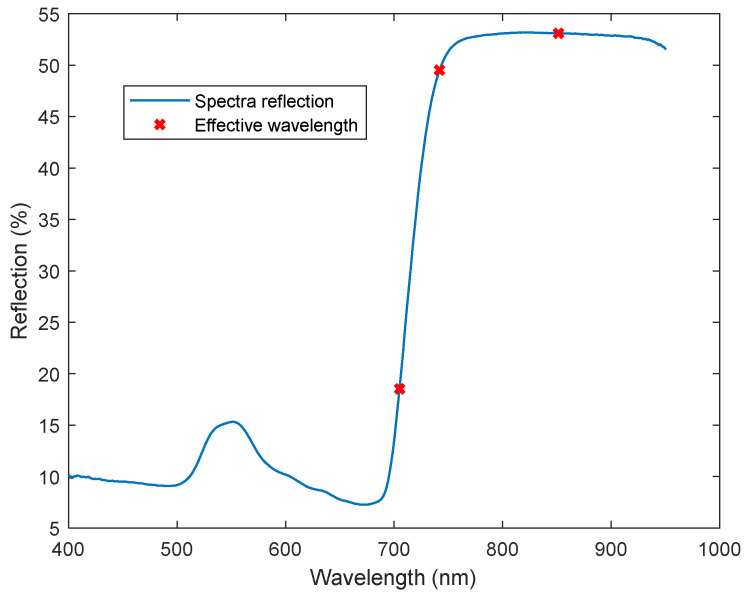
Extracted subset of the most informative wavelength using SPA.

**Figure 9:**
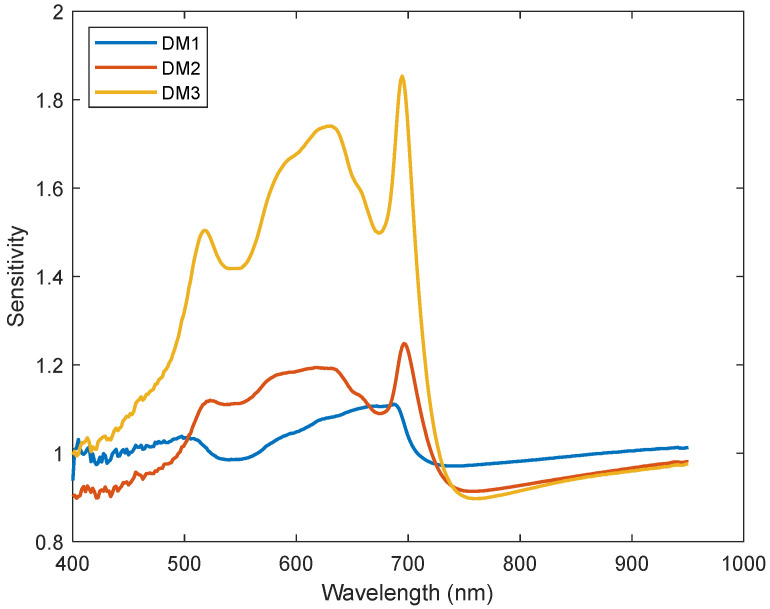
Sensitivity values of DM-infected leaves.

Following the application of the SPA algorithm to the spectral data, it has been revealed that 705 nm, 740 nm, and 850 nm wavelengths, as depicted in Figure 8, were informative wavelengths for downy mildew infection. In addition to the SPA calculations, sensitivity values were determined by dividing the mean reflectance value of diseased leaves by the mean reflectance value of healthy leaves at each specific wavelength. These sensitivity values served as an additional metric to investigate wavelengths that exhibited sensitivity to DM infection levels in contrast to those of healthy leaves. The sensitive values of DM-infected Mildis leaves are shown in Figure 9.

Figure 9 illustrates the sensitivity values for leaves infected with DM in comparison to healthy leaves. Noteworthy wavelengths include 450 nm, 550 nm, 630 nm, 680 nm, 705 nm, and 760 nm. These specific wavelengths exhibited notable local distinctions in sensitivity values across various levels of DM infection. Particularly, 705 nm and 760 nm emerged as pivotal points where sensitivity values end up indicating a significant shift across all DM infection levels.

In this study, three vegetation indices, *DMI1*, *DMI2*, and *DMI3*, were proposed to assess downy mildew levels. *DMI*1 Equation (2) aims to calculate the slope of reflection signals from 760 nm to 850 nm for leaves with different DM infection levels, since the reflection curves of different DM levels have different slopes from 760 nm to 900 nm. The *DMI2* method is a ratio-based approach designed to indicate that DM-infected leaves exhibit the largest sensitivity disparities at 705 nm (Equation (3)). This discrepancy is characterized by the highest sensitivity value for DM3, followed by a relatively smaller sensitivity value for DM2, and the smallest sensitivity value for DM1. As shown in Figure 9, the sensitivity values increase from 400 nm to 700 nm and from 760 nm to 950 nm for all DM infection levels. Based on this trend and the effective wavelengths obtained before, *DMI3* is formulated as shown in Equation (4).

(2) $$DMI1=\frac{R850-R760}{R850+R760}$$

(3) $$DMI2=\frac{R705}{R760}$$

(4) DMI3=(R850−R760)+(R680−R450)

**Figure 10:**
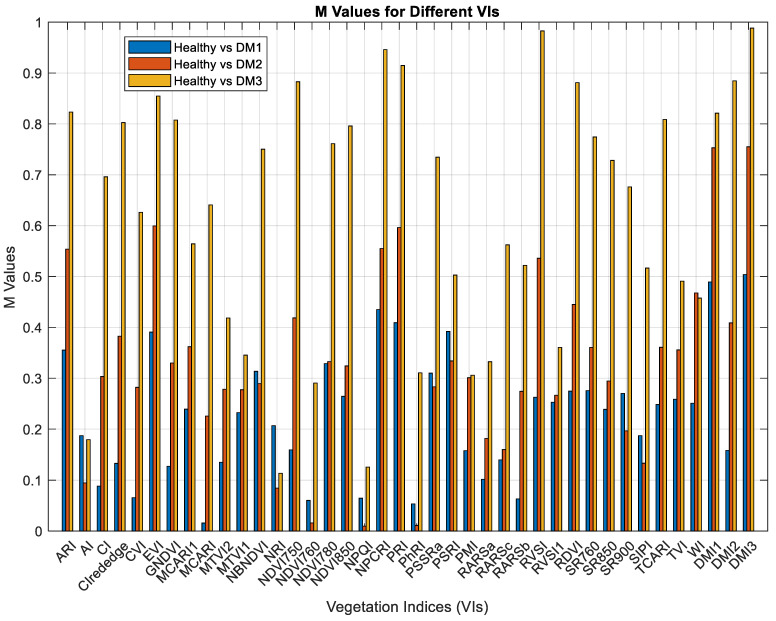
M values for VIs. Indices were calculated using data collected between 400 nm and 950 nm. (Note: The calculation of WI was an exception, as it involved data from 970 nm as needed in the formula.).

The M values (Figure 10) were calculated for each Vegetation Index in the context of contrasting healthy leaves with DM1, DM2, and DM3, respectively. In the initial downy mildew development stage (DM1), the vegetation indices *DMI3*, *DMI1*, and NPCRI exhibited the most substantial M values. In distinguishing healthy leaves from leaves with intermedium downy mildew infection (DM2), *DMI3*, *DMI1*, and *PRI* were observed as the most effective indices. In the final stage of downy mildew infection (DM3), *DMI3*, *RVSI*, and *NPCRI* were ranked as the leading VIs. Notably, *DMI3* consistently demonstrates the highest M values across all three distinct stages of DM infection. Consequently, it is apparent that the proposed Vegetation Index, *DMI3*, excels in discriminating leaves showing different levels of downy mildew infections from healthy sample leaves. 

**Figure 11:**
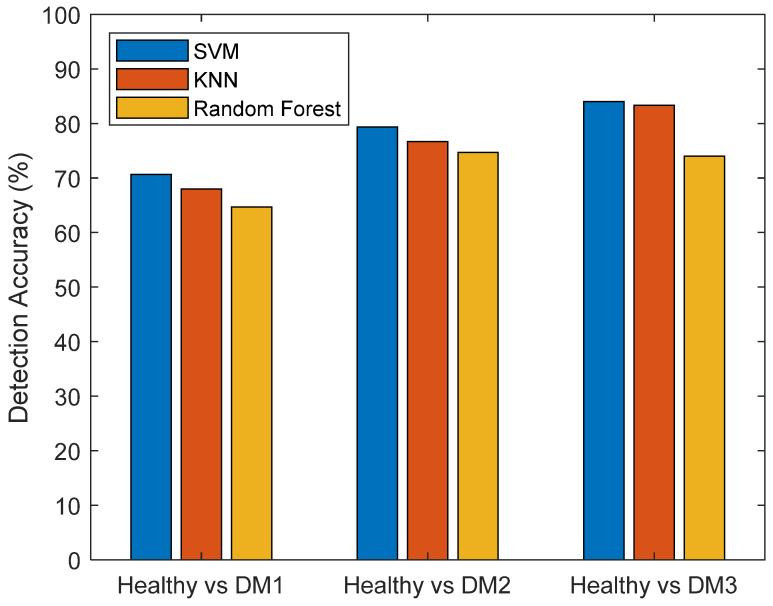
Classification accuracies for different downy mildew infection levels using SVM (Support Vector Machine), KNN (K-Nearest Neighbor), and Random Forest.

Three common machine learning classification algorithms, namely Support Vector Machine (SVM), K-Nearest Neighbor (KNN), and Random Forest, were employed for the categorization of data calculated using *DMI3*. A total of 400 data points, representing *DMI3*-calculated values against DM severity level labels, were randomly partitioned into training (320) and testing sets (80). The training dataset underwent a 5-fold cross-validation process, wherein it was subdivided into five folds, with four folds utilized for training and one for validation in each iteration. The optimal hyperparameters for these algorithms, as outlined in Table 3, were determined through a grid search. The prediction accuracy of the models on the testing dataset was determined by the ratio of correct predictions to the total number of predictions. The binary classification accuracies are visually presented in Figure 11. It is noteworthy that the SVM algorithm demonstrated the highest average classification accuracy, achieving 71.3% in distinguishing between healthy leaves and DM1, 80.7% in distinguishing between healthy leaves and DM2, and 85.3% in distinguishing between healthy leaves and DM3.

## 4. Discussion

This study applied SPA analysis to delineate wavelengths indicative of DM detection. The findings underscore the informative wavelengths at 705 nm, 740 nm, and 850 nm for DM identification. Moreover, sensitivity calculations reveal additional noteworthy wavelengths, specifically 450 nm, 550 nm, 630 nm, 680 nm, and 760 nm. Leveraging these informative wavelengths, three *DMI*s were introduced by this study. The calculations of M values for each Vegetation Index demonstrated that *DMI*3 consistently exhibits the highest M values across all three distinct stages of DM infection. This underscores the capacity of *DMI3* to differentiate DM-infected leaves from their healthy counterparts effectively. To categorize DM infection levels based on *DMI3* values, SVM, KNN, and Random Forest were trained utilizing a 5-fold cross-validation. The classification accuracies on the testing data set were reported as 71.3%, 80.7%, and 85.3% for distinguishing healthy leaves from early (DM1), intermedium (DM2), and severe (DM3) downy mildew-infected leaves using SVM. 

This study enhances our comprehension of the spectral responses exhibited by *Brassica* leaves in the presence of DM infection. It enables the identification of specific spectral indicators reflective of disease severity, potentially facilitating early detection and intervention strategies in *Brassica* crop management. Kanna and Kumar [8] achieved a 99.9% validation accuracy in automating the detection and classification of downy mildew on cauliflower. They employed deep transfer learning using regular RGB images [8]. The dataset consisted of leaf samples with severe disease infections, including large areas of diseased tissue [8]. Such large areas of infection led to distinct patterns of light and dark color on the leaves, which were, not surprisingly, successfully extracted using deep learning models and resulted in a 99.9% DM detection accuracy [8]. With the VIs developed in our study, we were not only limited to RGB bands but also included some invisible bands, which facilitated the identification of early DM symptoms (DM1) presented 3 to 4 days after infection, even before chlorotic tissue became visible. This technique could help breeders eliminate DM-susceptible breeding lines early on and expedite the DM resistance breeding process. The spectral data derived from *Brassica* leaves holds immense potential for the advancement of remote sensing techniques and the development of machine learning algorithms, which, in turn, can facilitate automated monitoring and management of DM in the context of *Brassica* breeding. These innovations pave the way for the evaluation of genotypic resistance to DM, providing a consistent, cost-effective, and efficient method for profiling downy mildew resistance in baby leaf salad green breeding lines. In addition, when applied in the production field, such an early DM detection technique may help growers determine when to apply preventative fungicides to avoid reductions in both crop yield and quality.

In future research endeavors, we will explore the integration of spectral imaging technologies and advanced machine learning algorithms, aiming to enable real-time, non-invasive DM monitoring in large-scale agricultural settings. Cheaper and special multispectral cameras will be developed based on the DMIs introduced, and the innovations hold the promise of continuous surveillance and rapid identification of DM outbreaks, contributing to more effective disease control measures, enabling high-throughput and cost-effective phenotyping methods to expedite the breeding process in *Brassica* production. Furthermore, the development of multi-modal sensing systems that combine RGB, thermal, and hyperspectral data will enable a comprehensive and precise assessment of DM severity. 

## Figures and Tables

**Table 1:** Defining levels of downy mildew infection in *Brassica* leaves.

Downy Mildew Disease Development Level	Description of Each Level
Healthy	No visual indication of infection on the leaves.
DM1	Pitted spots can be observed on the lower side of the leaves. The spots have not shown any chlorosis yet.
DM2	Pitted spots can be observed on both sides of the leaves. Chlorosis can be observed.
DM3	Lesions can be observed on the leaves.

**Table 2:** Vegetation Indices used in this study.

Vegetation Indices	Expressions	References
Anthocyanin Reflectance Index (ARI)	1/R550 − 1/R700	Gitelson, Merzlyak [26]
Aphid Index (AI)	(R740 − R887)/(R691 − R698)	Mirik, Michels [27]
Chlorohyll Index (CI green)	R840/R570 − 1	Gitelson, Gritz [28]
Chlorophyll Index Rededge (CIrededge)	R780/R705 − 1	Gitelson, Gritz [28]
Chlorophyll Vegetation Index (CVI)	R840 × R760/R550^2^	Vincini and Frazzi [29]
Enhanced Vegetation Index (EVI)	2.5 × ((R900 − R650)/(R900 + 6 × R650 − 7.5 × R500 + 1))	Huete, Didan [30]
Green NDVI (GNDVI)	(R850 − R580)/(R850 + R580)	Gitelson and Merzlyak [31]
Modified Chlorophyll Absorption in Reflectance Index (mCARI1)	1.2 × (2.5 × (R761 − R651) − 1.3 × (R761 − R580))	Haboudane, Miller [32]
Modified Chlorophyll Absorption Ratio Index (mCARI)	(R700 − R670) − 0.2 × (R700 − R550) × ((R700/R670) − 1)	Daughtry, Walthall [33]
Modified Triangle Vegetation Index 2 (MTVI2)	1.5 × (1.2 × (R800 − R550) − 2.5 × (R670 − R550)) × (2 × R800 + 1)^2^ − (6 × R800 – 5 × R670) − 0.5	Haboudane, Miller [32]
Modified Triangle Vegetation Index 1 (MTVI1)	1.2 × (1.2 × (R800 − R550) − 2.5 × (R670 − R550))	Haboudane, Miller [32]
Narrow Band Normalized Difference Vegetation Index (NBNDVI)	(R850 − R680)/(R850 + R680)	Thenkabail, Smith [34]
Nitrogen Reflectance Index (NRI)	(R570 − R670)/(R570 + R670)	Filella, Serrano [35]
Normalized Difference Vegetation Index 750 (NDVI 750)	(R750 − R705)/(R750 + R705)	Raun, Solie [36]
Normalized Difference Vegetation Index 760 (NDVI 760)	(R761 − R450)/(R761 + R450)	Raun, Solie [36]
Normalized Difference Vegetation Index 780 (NDVI 780)	(R780 − R670)/(R780 + R670)	Raun, Solie [36]
Normalized Difference Vegetation Index 850 (NDVI 850)	(R850 − R651)/(R850 + R651)	Raun, Solie [36]
Normalized Phaeophytization Index (NPQI)	(R415 − R435)/(R415 + R435)	Barnes, Balaguer [37]
Normalized Pigment Chlorophyll Ratio Index (NPCRI)	(R680 − R430)/(R680 + R430)	Penuelas, Frederic [38]
Photochemical Reflectance Index (PRI)	(R570 − R531)/(R570 + R531)	Penuelas, Llusia [39]
Photosynthetic Radiation (PhRI)	(R550 − R531)/(R550 + R531)	Gamon, Peñuelas [40]
Pigment Specific Simple Ratio (PSSRa)	R800/R680	Blackburn [41]
Plant Senescence Reflectance Index (PSRI)	(R680 − R500)/R750	Merzlyak, Gitelson [42]
Powdery Mildew Index (PMI)	(R515 − R698)/(R515 + R698) − 0.5 × R738	Huang, Guan [43]
Ratio Analysis of Reflectance Spectral Chlorophyll-a (RARSa)	R675/R700	Chappelle, Kim [44]
Ratio Analysis of Reflectance Spectra (RARSc)	R760/R500	Chappelle, Kim [44]
Ratio Analysis of Reflectance Spectral Chlorophyll-b (RARSb)	R675/(R700 × R650)	Chappelle, Kim [44]
Red Edge Vegetation Stress Index (RVSI)	(R712 + R752)/2 − R732	Merton and Huntington [45]
Red-Edge Vegetation Stress Index1 (RVSI1)	(R650 + R750)/2 − R733	Merton [46]
Renormalized Difference Vegetation Index (RDVI)	$(R760-R650)/\sqrt{R760+R650}$	Roujean and Breon [47]
Simple Ratio Index (SR760)	R761/R650	Jordan [48]
Simple Ratio Index (SR850)	R850/R650	Jordan [48]
Simple Ratio Index (SR900)	R900/R680	Jordan [48]
Structure Insensitive Pigment Index (SIPI)	(R800 − R445)/(R800 + R680)	Penuelas, Frederic [38]
Transform chlorophyll absorption in reflectance index (TCARI)	3 × ((R740 − R651) − 0.2 × (R740 − R580) × (R740/R651))	Haboudane, Miller [49]
Triangle Vegetation Index (TVI)	0.5 × (120 × (R750 − R550) – 200 × (R670 − R550))	Broge and Leblanc [50]
Water Index (WI)	R900/R970	Penuelas, Pinol [51]

**Table 3:** Hyperparameters used in the classification algorithms.

	Accuracy			Model Hyperparameters
	Healthy vs. DM1	Healthy vs. DM2	Healthy vs. DM3	
SVM	71.3%	80.7%	85.3%	Kerna function: Gaussian Kernel scale: 0.25 Box constraint: 1 Standardized data
KNN	67.3%	78.7%	84.1%	Number of neighbors: 10 Distance metric: Euclidean Standardized data
Random Forest	64.1%	74.2%	74.6%	Number of decision trees: 100 Number of predictors to sample: 2 Min. leaf size: 1

Note: DM = downy mildew, Support Vector Machine = SVM, and KNN = K-Nearest Neighbor.

## Data Availability

The raw data supporting the conclusions of this article will be made available by the authors on request.
